# Survival After Childhood Cancer–Social Inequalities in High-Income Countries

**DOI:** 10.3389/fonc.2018.00485

**Published:** 2018-10-31

**Authors:** Hanna Mogensen, Karin Modig, Giorgio Tettamanti, Friederike Erdmann, Mats Heyman, Maria Feychting

**Affiliations:** ^1^Unit of Epidemiology, Institute of Environmental Medicine, Karolinska Institutet, Stockholm, Sweden; ^2^Section of Environment and Radiation, International Agency for Research on Cancer (IARC), Lyon, France; ^3^Childhood Cancer Research Group, Danish Cancer Society Research Center, Copenhagen, Denmark; ^4^Childhood Cancer Research Unit, Department of Women's and Children's Health, Karolinska Institutet and Karolinska University Hospital, Stockholm, Sweden

**Keywords:** childhood neoplasms, leukemia, nervous system neoplasms, socioeconomic factors, survival, review

## Abstract

Despite substantial improvements in survival from childhood cancer during the last decades, there are indications that survival rates for several cancer types are no longer improving. Moreover, evidence accumulates suggesting that socioeconomic and sociodemographic factors may have an impact on survival also in high-income countries. The aim of this review is to summarize the findings from studies on social factors and survival in childhood cancer. Several types of cancer and social factors are included in order to shed light on potential mechanisms and identify particularly affected groups. A literature search conducted in PubMed identified 333 articles published from December 2012 until June 2018, of which 24 fulfilled the inclusion criteria. The findings are diverse; some studies found no associations but several indicated a social gradient with higher mortality among children from families of lower socioeconomic status (SES). There were no clear suggestions of particularly vulnerable subgroups, but hematological malignancies were most commonly investigated. A wide range of social factors have been examined and seem to be of different importance and varying between studies. However, potential underlying mechanisms linking a specific social factor to childhood cancer survival was seldom described. This review provides some support for a relationship between lower parental SES and worse survival after childhood cancer, which is a finding that needs further attention. Studies investigating predefined hypotheses involving specific social factors within homogenous cancer types are lacking and would increase the understanding of mechanisms involved, and allow targeted interventions to reduce health inequalities.

## Introduction

From low survival rates in the 1970's and earlier, overall 5 years survival from childhood cancer is now exceeding 80% in most of Europe (1, 2). Nonetheless, despite these advances a significant number of children with cancer fail to reach this milestone, with varying proportions according to cancer type (2). Moreover, reports from the US and Europe indicate that survival improvements for several childhood cancer types have leveled off during recent years (2, 3). At the same time, evidence accumulates suggesting that socioeconomic and sociodemographic factors may be associated with survival even in high-income countries where children are presumed to have equal access to health care services, see for example (4–7). This does not only highlight a potential inequality that needs attention, but might imply a possibility of improving childhood cancer survival rates overall, by addressing this potential gap. However, even though several studies support an association between higher parental socioeconomic status (SES) and better survival, findings differ between countries, cancer types, and SES indicator studied. Some of the differences might be explained by inconsistent methodology between studies, but might also indicate different mechanisms in which parental SES affects survival. For example, differences in treatment and prognosis between cancer types are likely to influence.

Gupta et al. (8) conducted a systematic review evaluating the association between SES and childhood cancer survival, including studies published until 2012. This review indicated that in high income countries, parental income is not the driver of the association but instead other SES indicators such as education, having insurance, or place of residence seemed to be of importance (8). However, parental income was only assessed in few studies. Since 2012, there have been several studies examining the association between parental SES and survival from childhood cancer in high income countries, and these are the focus of the current review.

The objectives of this review are (i) to summarize the findings from studies on social factors and survival from childhood cancer in high-income countries, by cancer type, and (ii) to elucidate the role of different socioeconomic and sociodemographic factors (parental education, income, social status based on occupation, cohabitation, and marital status, place of residence, number of siblings, and birth order) on the association, in order to shed light on potential mechanisms and to identify particularly affected groups.

## Methods

A literature search was conducted in PubMed (the 15th of June 2018) and included articles published from December 2012 until mid-June 2018, this corresponds to the time period following the previous systematic review (8). The search included terms related to cancer, survival, children, and socioeconomic and sociodemographic factors (for details see Supplementary Table 1). Titles, abstracts and full-texts were screened for relevance by one of the authors (HM). *A priori* defined inclusion criteria were: non-ecological, original articles, conducted in high-income countries, that restricted analyses to childhood cancer of any type and assessed the association with at least one socioeconomic or sociodemographic factor in relation to overall survival, relative survival or event-free survival. Studies focusing on cancer types primarily affecting adults were excluded. Included individual measures of SES were parental education, parental income, parental occupation, parental cohabitation and marital status, place of residence, number of siblings and birth order. Also studies using area-based measures of SES were included. No restrictions on language were applied.

From all included studies information on setting, cancer diagnoses, study size and diagnostic period, source of identification of cancer cases, socioeconomic, and sociodemographic measurements of relevance, outcome of relevance, as well as main results of interest were extracted by one of the authors (HM). Also results of the association between specific social factors and survival, from each of the included studies, were extracted and included in tables by cancer type, most often in terms of hazard ratios (HR) and corresponding 95% confidence intervals (CI). Similar to the previous review in this field (8), no quantitative meta-analysis was considered due to the diversity of social factors included, but findings were summarized in a narrative synthesis.

## Results

**Figure 1:**
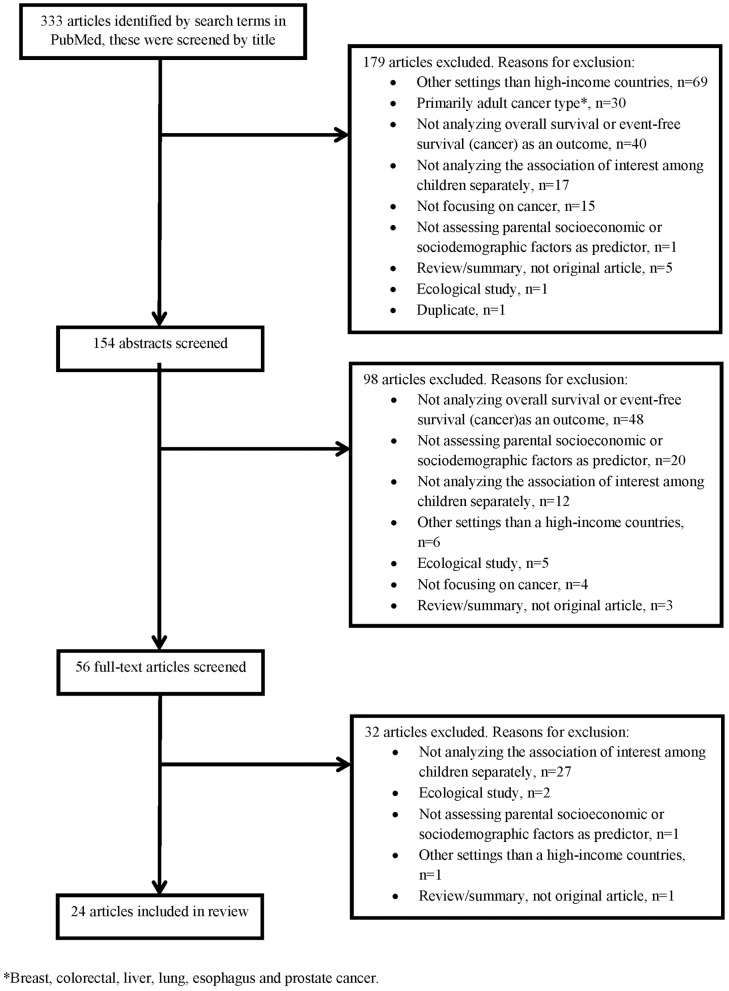
Flow chart of title, abstract and full-text screening.

Twenty-four of the 333 articles identified by the literature search met the inclusion criteria and were included in this review (Table 1). Exclusions were made based on titles (179 articles), abstracts (98 articles), and full-texts (32 articles), Figure 1 shows the reasons for exclusion in a flow diagram. Tables 2A,B summarize the main results of the included studies.

**Table 1:** Description of included studies.

**References**	**Setting**	**Included diagnoses**	**Study size and diagnostic period**	**Source of identification of cancer cases**	**Socioeconomic and sociodemographic measurements of relevance**	**Outcome of relevance**
(9)	Denmark	All diagnoses combined; hematological malignancies– ALL, CNS tumors, non-CNS solid tumors	3,797 children, diagnosed < 20 years old during 1990-2009	Danish cancer registry	Individual level: Maternal and paternal education, maternal income, parents' cohabitation status, and number of full siblings < 19 years, based on registries	Overall survival
(5)	Switzerland	All diagnoses combined; leukemia- ALL, lymphoma, CNS tumors, bone and soft tissue tumors, embryonal tumors	1,602 children, diagnosed < 16 years old during 1991– 2006	Swiss childhood cancer registry	Individual level: Maternal and paternal education, and living conditions (number of rooms per person, living space), based on census. Area-based: SES-index	5 year cumulative mortality
(6)	Sweden	All diagnoses combined; leukemia- ALL, tumors of the nervous system- brain tumors, lymphoma	4,723 children, diagnosed 1-14 years old during 1991–2010	Swedish cancer registry	Individual level: Parental education, and household income, based on registries	Overall survival, follow-up for maximum 10 years
(7)	Finland	All diagnoses combined; ALL and LBL, CNS tumors, all other malignant neoplasms	4,437 children diagnosed < 20 years old during 1990–2009	Finnish cancer registry	Individual level: Combined parental income, highest parental education, maternal and paternal employment status, based on registers	Cause specific mortality (death from primary cancer) and childhood cancer specific survival, follow-up for maximum 5 years
(10)	Northern England	Leukemia; ALL, acute non-lymphocytic leukemia	1,007 children, diagnosed 0-14 years old during 1968-2010	Northern region young persons malignant disease registry	Individual level: Paternal occupational social class, based on birth certificate	Overall mortality
(11)	U.S	Hematologic malignancies, CNS tumors, solid tumors	36,337 children, diagnosed 0-19 years old during 1992–2011	SEER	Area-based: Poverty, education, unemployment, language isolation, foreign-born, and income, based on census	Death within one month of diagnosis
(12)	West Germany	ALL	647 children, diagnosed < 15 years old during 1992–1994	German childhood cancer registry	Individual level: Maternal and paternal education, family income, and residential area, based on telephone interviews (response rate 82%)	Overall survival and event-free survival, follow-up for maximum 10 years
(13)	Greece	ALL, AML	994 children, diagnosed 0–14 years old during 1996–2010	Nationwide registry for childhood hematological malignancies	Individual level: Parental marital status, parental socioprofessional category, maternal education, number of children, place of living, and travel distance, based on questionnaires	Overall mortality
(14)	West Germany	ALL	647 children, diagnosed < 15 years old during 1992–1994	The German childhood cancer registry	Individual level: Birth order, number of siblings, place of residence, based on questionnaires (response rate 82%)	Overall survival and event-free survival, follow-up for maximum 10 years
(15)	Canada	ALL	1,541 children diagnosed < 18 years old during 1995–2011	Pediatric oncology group of ontario networked information system	Individual level: Rurality, distance from tertiary center Area-based: Neighborhood income, based on census	Event-free survival
(16)	California, U.S.	ALL	9,295 children diagnosed 0–19 years old during 1988–2011	California cancer registry	Area-based: Neighborhood SES, based on census	Overall survival
(17)	Texas & Florida, U.S.	ALL	4,719 children diagnosed 1–18 years old during 1995–2008	Florida cancer data system and the Texas cancer registry	Area-based: Neighborhood-level poverty rate, based on census	Overall survival
(18)	U.S.	ALL	8,516 children, diagnosed < 19 years old during 1999–2009	Pediatric health information system	Area-based: ZIP-code based median household income, based on census	Inpatient mortality, death during the induction period. The children were followed from the first day of chemotherapy (in inpatient care) until maximum 60 days
(19)	U.S.	AML	3,651 children diagnosed 0–19 years old during 1973–2012	SEER	Area-based: SES factors and clusters constructed from 23 socioeconomic variables, based on census	Overall mortality
(20)	Denmark	Hematological malignancies; ALL, AML, non-Hodgkin lymphoma	1,819 children diagnosed < 20 years old during 1973–2006	Danish cancer registry	Individual level: Birth order, number of full and half siblings, place of residence, based on registers	Overall survival, follow-up for maximum 10 years
(21)	Ontario, Canada	Lymphoma; Hodgkin lymphoma, non-Hodgkin lymphoma	692 children diagnosed 0–14 years old during 1985–2006	Pediatric oncology group of ontario networked information system database	Area-based: Neighborhood income and material deprivation, based on census	Overall survival and event-free survival
(22)	Denmark	CNS tumors; astrocytomas and other gliomas, embryonal CNS tumors	1,261 children diagnosed < 20 years old during 1973–2006	Danish Cancer Registry	Individual level: Birth order, number of siblings, number of children living in the household, place of residence, parental cohabitation, maternal education, based on registries	Overall survival, follow-up for maximum 10 years
(23)	Texas, U.S.	Primary CNS solid tumors	2,421 children diagnosed < 19 years during 1995 and 2009	Texas cancer registry	Individual level: Driving distance to cancer center Area-based: Block level SES index, based on census	Overall survival
(24)	Texas, U.S.	Non-CNS solid tumor	4,603 children diagnosed < 19 years old during 1995-2009	Texas cancer registry	Individual level: Driving distance to cancer center Area-based: Block level SES index, based on census	Overall survival
(25)	Texas, U.S.	Melanoma	235 children diagnosed < 19 years old during 1995–2009	Texas cancer registry	Individual level: Driving distance to cancer center Area-based: Block level SES index, based on census	Overall survival
(26)	Northern England	Renal tumors combined: Wilms tumors	209 patients (183 in SES analysis) diagnosed 0–24 years old during 1968–2012 Multivariate analyses are performed only among children diagnosed 0–14 years old with Wilms' tumor	Northern region young persons' malignant disease registry	Individual level: Paternal occupational social class based on birth certificate	Overall survival
(27)	U.S.	Well-differentiated thyroid cancer	9,585 children < 22 years old from the register 1998–2012	National cancer database	Area-based: ZIP-code based median income and education, categorized by census data	Overall mortality
(28)	U.S.	Disseminated Langerhans cell histiocytosis	145 children diagnosed 0–19 years old during 2000–2009	SEER	Area-based: Crowding, educational attainment, poverty level, and rural/urban county, based on census	5 year relative survival
(29)	U.S.	Retinoblastoma	830 children 0–9 years old diagnosed 2000–2010	SEER	Area-based: County-level poverty, educational attainment, crowding, unemployment, immigration, language isolation, and SES-index, based on census	5 year relative survival

*ALL, Acute lymphoblastic leukemia; AML, Acute myeloid leukemia; CNS, Central nervous system; LBL, Lymphoblastic lymphoma; SEER, The Surveillance, Epidemiology, and End Results*.

**Table 2A:** Main results of the included studies regarding the associations between socioeconomic factors and survival.

**References**	**Education**		**Income**		**Employment/occupation**		**Area-based SES indicator**	
		**HR^a^ (95% CI)**		**HR^a^ (95% CI)**		**HR^a^ (95% CI)**		**HR^a^ (95% CI)**
**ALL DIAGNOSES COMBINED**								
(9)	**Maternal**		**Maternal, quartiles**				
	Basic	1 (ref)	1st (lowest)	1 (ref)			
	Vocational	0.93 (0.75–1.15)	2nd	1.01 (0.84–1.21)			
	Higher	0.88 (0.69–1.13)	3rd	0.92 (0.75–1.14)			
	Unknown	1.05 (0.74–1.49)	4th	0.84 (0.66–1.08)			
	**Paternal**					
	Basic	1 (ref)					
	Vocational	0.90 (0.74–1.10)					
	Higher	0.89 (0.70–1.13)					
	Unknown	1.05 (0.75–1.46)					
(5)	**Maternal**						**SES index, tertiles**	
	Compulsory	1 (ref)					Lower	1 (ref)
	Secondary	0.81 (0.65–1.02)					Medium	0.93 (0.71–1.20)
	Tertiary	0.67 (0.45–0.98)					Upper	0.95 (0.73–1.24)
	**Paternal**					
	Compulsory	1 (ref)					
	Secondary	0.85 (0.64–1.11)					
	Tertiary	0.72 (0.53–0.98)					
					
(6)	**Parental**		**Household, quartiles**				
	Postsecondary	1 (ref)	4th (highest)	1 (ref)			
	Upper secondary	1.17 (1.00–1.38)	3rd	0.85 (0.69–1.04)			
	Compulsory or less	1.28 (1.03–1.59)	2nd	0.96 (0.79–1.18)			
			1st	1.03 (0.85–1.26)			
(7)	**Parental**		**Combined parental, quartiles**		**Maternal employment status**		
	Primary or less	1 (ref)	1st (lowest)	1 (ref)	Employed	1 (ref)	
	Secondary	1.00 (0.79–1.27)	2nd	0.83 (0.63–1.09)	Unemployed	0.84 (0.64–1.09)	
	Post-secondary	0.84 (0.66–1.06)	3rd	0.76 (0.58–1.00)	Student	1.39 (0.98–1.98)	
			4th	0.68 (0.52–0.89)	Pensioner	0.91 (0.51–1.62)	
			Information missing	0.93 (0.61–1.41)	Other non-working	1.10 (0.90–1.35)	
			Structural missing	0.78 (0.53–1.15)	Information missing	1.61 (0.98–2.66)	
					**Paternal employment status**	
					Employed	1 (ref)	
					Unemployed	1.14 (0.89–1.47)	
					Student	1.31 (0.80–2.15)	
					Pensioner	1.00 (0.65–1.54)	
					Other non-working	1.41 (0.87–2.29)	
					Information missing	1.26 (0.91–1.75)	
**HEMATOLOGICAL CANCERS**								
(9)	**Maternal**	**Maternal, quartiles**			
	Basic	1 (ref)	1st (lowest)	1 (ref)			
	Vocational	1.05 (0.71–1.56)	2nd	1.17 (0.85–1.60)			
	Higher	1.10 (0.70–1.73)	3rd	0.81 (0.55–1.20)			
	Unknown	1.00 (0.54–1.86)	4th	0.82 (0.53–1.28)			
	**Paternal**					
	Basic	1 (ref)					
	Vocational	1.14 (0.78–1.66)					
	Higher	0.95 (0.60–1.50)					
	Unknown	1.94 (1.07–3.49)					
(11)							**Education**^b^	
							Univariate	
							Advantaged	1 (ref)
							Disadvantaged	1.43 (1.12–1.83)
							**Income**^b^
							Univariate	
							Advantaged	1 (ref)
							Disadvantaged	1.66 (1.30–2.12)
							Adjusted	
							Advantaged	1 (ref)
							Disadvantaged	1.51 (1.07–2.14)
**LEUKEMIA**								
(5)	**Maternal**						**SES index, tertiles**	
	Compulsory	1 (ref)					Lower	1 (ref)
	Secondary	1.06 (0.69–1.61)					Medium	0.90 (0.56–1.42)
	Tertiary	1.05 (0.58–1.91)					Upper	1.06 (0.66–1.71)
	**Paternal**					
	Compulsory	1 (ref)					
	Secondary	1.39 (0.81–2.38)					
	Tertiary	1.45 (0.82–2.58)					
(6)	**Parental**		**Household, quartiles**				
	Postsecondary	1 (ref)	4th (highest)	1 (ref)			
	Upper secondary	1.28 (0.95–1.74)	3rd	1.05 (0.72–1.53)			
	Compulsory or less	1.39 (0.93–2.08)	2nd	1.06 (0.72–1.56)			
			1st	1.22 (0.83–1.78)			
(10)					**Paternal social class based on occupation**		
					I/II (most advantaged)	1 (ref)	
					IIIN/M	1.66 (1.20–2.29)	
					IV/V	1.96 (1.35–2.86)	
**ALL and LBL**								
(7)	**Parental**		**Combined parental, quartiles**		**Maternal employment status**		
	Primary or less	1 (ref)	1st (lowest)	1 (ref)	Employed	1 (ref)	
	Secondary	1.12 (0.66–1.88)	2nd	0.91 (0.49–1.71)	Unemployed	0.66 (0.35–1.28)	
	Post-secondary	0.82 (0.48–1.40)	3rd	0.76 (0.40–1.44)	Student	2.02 (0.88–4.64)	
			4th	0.86 (0.47–1.57)	Pensioner	0.50 (0.07–3.58)	
			Information missing	0.60 (0.18–2.08)	Other non-working	1.24 (0.82–1.89)	
			Structural missing	1.08 (0.45–2.60)	Information missing	1.72 (0.54–5.50)	
					**Paternal employment status**		
					Employed	1 (ref)	
					Unemployed	1.43 (0.85–2.42)	
					Student	0.85 (0.21–3.46)	
					Pensioner	0.81 (0.26–2.59)	
					Other non-working	1.20 (0.38–3.80)	
					Information missing	1.13 (0.50–2.58)	
**ALL**								
(6)	**Parental**		**Household, quartiles**				
	Postsecondary	1 (ref)	4th (highest)	1 (ref)			
	Upper secondary	1.26 (0.86–1.87)	3rd	1.20 (0.74–1.94)			
	Compulsory or less	0.98 (0.55–1.74)	2nd	0.95 (0.57–1.59)			
			1st	1.24 (0.76–2.04)			
(10)					**Paternal social class based on occupation**		
					I/II (most advantaged)	1 (ref)	
					IIIN/M	1.68 (1.20–2.36)	
					IV/V	1.86 (1.24–2.77)	
(12)	**Maternal**		**Family**			
	No degree	1.07 (0.38–3.04)	< 2,000 DM	1.21 (0.60–2.44)			
	Low degree	1 (ref)	2,000–4,000 DM	1 (ref)			
	Intermediate degree	0.69 (0.41–1.17)	4,000–6,000 DM	0.80 (0.47–1.38)			
	High degree	0.92 (0.52–1.62)	6,000–8,000 DM	1.27 (0.52–3.06)			
			>8,000 DM	1.11 (0.37–3.29)			
(13)	**Maternal**				**Parental socioprofessional category**		
	Four categories, per increase of one level	1.11 (0.90–1.37)			Three categories, per increase of one level	0.71 (0.54-0.94)	
(15)							**Neighborhood median income, quintiles**	
							1st (lowest)	Ref
							2nd	0.93 (0.62–1.40)
							3rd	1.03 (0.69–1.54)
							4th	1.09 (0.74–1.62)
							5th	1.09 (0.72–1.64)
(16)							**Neighborhood SES, quintiles**	
							1st (lowest 20%)	1.39 (1.18–1.64)
							2nd	1.15 (0.97–1.35)
							3rd	1.13 (0.95–1.33)
							4th	1.17 (0.99–1.39)
							5th	1 (ref)
(17)							**Neighborhood-level poverty rate (% of households living in poverty)**	
							0- < 5	1 (ref)
							5- < 20	1.29 (1.03–1.61)
							20–100	1.80 (1.41–2.30)
(18)							**Median household income based on ZIP-code**	
							Univariate	
							For every $10,000/year increase	0.95 (0.84–1.07)
**AML**								
(10)					**Paternal social class based on occupation**		
					Unadjusted		
					I/II (most advantaged)	1 (ref)	
					IIIN/M	1.47 (0.57–3.80)	
					IV/V	2.05 (0.77–5.44)	
(13)	**Maternal**				**Parental socioprofessional category**		
	Four categories, per increase of one level	0.99 (0.65-1.52)			Three categories, per increase of one level	0.89 (0.49-1.62)	
(19)							**SES factors and clusters**	
							One unit increase in the average score of each factor	
							Factor 1 (economic and educational disadvantage)	1.07 (1.02–1.12)
							Factor 2 (immigration)	0.99 (0.94–1.04)
							Factor 3 (housing instability)	1.05 (1.00–1.10)
							Factor 4 (low rates of moving within the state)	0.98 (0.93–1.03)
							Clusters were formed based on factors and compared. Lowest AML mortality was seen in Cluster 1 which reflected low Factor 1, 2, & 3.	
**LYMPHOMA**								
(5)	**Maternal**						**SES index, tertiles**	
	Compulsory	1 (ref)					Lower	1 (ref)
	Secondary	0.71 (0.30–1.66)					Medium	1.09 (0.38–3.09)
	Tertiary	0.40 (0.05–3.19)					Upper	1.51 (0.55–4.16)
	**Paternal**					
	Compulsory	1 (ref)					
	Secondary	0.40 (0.16–1.02)					
	Tertiary	0.26 (0.08–0.85)					
(6)	**Parental**		**Household, quartiles**				
	Postsecondary	1 (ref)	4th (highest)	1 (ref)			
	Upper secondary	1.35 (0.69–2.64)	3rd	0.67 (0.28–1.56)			
	Compulsory or less	1.13 (0.46–2.77)	2nd	1.36 (0.63–2.94)			
			1st	1.37 (0.62–3.02)			
(21)							**Material deprivation, quintiles**	
							Hodgkin lymphoma	
							1st	0.63 (0.13–3.17)
							2nd	1.16 (0.38–3.52)
							3rd	1.41 (0.52–3.83)
							4th	0.99 (0.30–3.27)
							5th (least deprived)	1 (ref)
							Non-hodgkin lymphoma	
							1st	1.26 (0.49–3.24)
							2nd	1.45 (0.57–3.68)
							3rd	1.37 (0.57–3.29)
							4th	2.33 (1.03–5.30)
							5th (least deprived)	1 (ref)
**CNS TUMORS/TUMORS OF THE NERVOUS SYSTEM**								
(9)	**Maternal**		**Maternal, quartiles**				
	Basic	1 (ref)	1st (lowest)	1			
	Vocational	1.20 (0.79–1.82)	2nd	0.92 (0.66–1.28)			
	Higher	1.17 (0.73–1.89)	3rd	0.84 (0.58–1.22)			
	Unknown	1.42 (0.73–2.78)	4th	0.86 (0.55–1.34)			
	**Paternal**					
	Basic	1 (ref)					
	Vocational	0.82 (0.58–1.17)					
	Higher	0.89 (0.58–1.36)					
	Unknown	0.73 (0.39–1.36)					
(5)	**Maternal**						**SES index, tertiles**	
	Compulsory	1 (ref)					Lower	1 (ref)
	Secondary	0.59 (0.39–0.90)					Medium	0.70 (0.43–1.15)
	Tertiary	0.52 (0.26–1.05)					Upper	0.71 (0.44–1.15)
	**Paternal**					
	Compulsory	1 (ref)					
	Secondary	0.62 (0.38–1.01)					
	Tertiary	0.48 (0.28–0.81)					
(6)	**Parental**		**Household, quartiles**				
	Postsecondary	1 (ref)	4th (highest)	1 (ref)			
	Upper secondary	0.99 (0.77–1.26)	3rd	0.78 (0.57–1.07)			
	Compulsory or less	1.25 (0.90–1.73)	2nd	0.87 (0.64–1.19)			
			1st	1.07 (0.79–1.43)			
(7)	**Parental**		**Combined parental, quartiles**		**Maternal employment status**		
	Primary or less	1 (ref)	1st (lowest)	1 (ref)	Employed	1 (ref)	
	Secondary	0.75 (0.48–1.17)	2nd	0.62 (0.35–1.07)	Unemployed	0.77 (0.45–1.32)	
	Post-secondary	0.69 (0.44–1.08)	3rd	0.92 (0.54–1.55)	Student	1.47 (0.81–2.67)	
			4th	0.69 (0.40–1.18)	Pensioner	0.97 (0.31–3.06)	
			Information missing	1.16 (0.51–2.63)	Other non-working	0.98 (0.67–1.43)	
			Structural missing	0.56 (0.25–1.28)	Information missing	1.70 (0.54–5.38)	
					**Paternal employment status**		
					Employed	1 (ref)	
					Unemployed	1.01 (0.61–1.67)	
					Student	1.34 (0.59–3.04)	
					Pensioner	1.10 (0.48–2.52)	
					Other non-working	2.11 (0.86–5.16)	
					Information missing	1.38 (0.70–2.72)	
(11)							**Education**^b^	
							Univariate	
							Advantaged	1 (ref)
							Disadvantaged	1.30 (0.94–1.79)
							**Income**^b^	
							Univariate	
							Advantaged	1 (ref)
							Disadvantaged	1.19 (0.87–1.65)
(22)	**Maternal**					
	Short	0.91 (0.68–1.23)					
	Medium	1.10 (0.87–1.39)					
	Higher	1 (ref)					
(23)							**SES index, quartiles**	
							< 25%	1.13 (0.90–1.43)
							25–50%	1.17 (0.93–1.48)
							51–75%	0.97 (0.77–1.22)
							>75%	1 (ref)
**OTHER TUMORS**								
(9)	**Maternal**		**Maternal, quartiles**				
	Basic	1 (ref)	1st (lowest)	1 (ref)			
	Vocational	0.79 (0.56–1.11)	2nd	0.88 (0.65–1.20)			
	Higher	0.66 (0.44–0.99)	3rd	1.11 (0.80–1.55)			
	Unknown	0.88 (0.48–1.63)	4th	0.81 (0.53–1.24)			
	**Paternal**					
	Basic	1 (ref)					
	Vocational	0.81 (0.59–1.11)					
	Higher	0.97 (0.65–1.43)					
	Unknown	0.87 (0.45–1.54)					
(11)							**Education**^b^	
							Univariate	
							Advantaged	1 (ref)
							Disadvantaged	1.05 (0.73-1.49)
							**Income**^b^	
							Univariate	
							Advantaged	1 (ref)
							Disadvantaged	1.20 (0.84-1.71)
(24)							**SES index, quartiles**	
							< 25%	1.1 (0.9–1.3)
							25–50%	1.0 (0.8–1.2)
							50–75%	1.0 (0.8–1.2)
							>75%	1 (ref)
(25)							**SES index, quartiles**	
							< = 25%	2.8 (0.8-9.6)
							26–50%	1.6 (0.4-6.3)
							51–75%	0.9 (0.3-3.6)
							>75%	1 (ref)
(26)					**Paternal social class based on occupation**		
					Renal tumors (age 0–24), univariate		
					I/II (most affluent)	1 (ref)	
					IIIN/M	1.18 (0.60–2.30)	
					IV/V	1.17 (0.53–2.62)	
					Wilms' tumor (age 0-14), multivariate		
					I/II (most affluent)	1 (ref)	
					IIIN/M	1.12 (0.48–2.59)	
					IV/V	1.47 (0.55–3.91)	
(27)							**Median income and education, quartiles**	
							No estimates reported. Overall survival curves show no statistical significant differences between the groups.	
(28)							*5 year relative survival rates (%)*	
							**Percent low educated**^b^	
							< = 16.6	97.0 (78.0–99.6)
							>16.6	87.8 (79.1–93.0) (*p-*value 0.156)
							**Percent below poverty level**^b^	
							< = 8.85	94.3 (85.0–97.9)
							>8.85	85.6 (73.7–92.3) (*p*-value 0.123)
(29)							*5 year relative survival rates (%)*	
							**Poverty level**^b^	
							Low	98.8
							High	96.4 (*p*-value 0.054)
							**Education level**^b^	
							High	98.5
							Low	96.8 (*p*-value 0.154)
							**Socioeconomic index**^b^	
							Low	98.9
							High (more disadvantages counties)	96.5 (*p*-value 0.070)

a *Adjusted results if not otherwise stated. RR instead of HR is presented in some studies*.

b Several area-based indicators were reported in the study but only measures corresponding to education, income and SES index are included in this table

*ALL, Acute lymphoblastic leukemia; AML, Acute myeloid leukemia; CI, Confidence interval; CNS, Central nervous system; HR, Hazard ratio; LBL, Lymphoblastic lymphoma; SEER, The Surveillance, Epidemiology, and End Results*.

**Table 2B:** Main results of the included studies regarding the associations between sociodemographic factors and survival.

**References**	**Siblings and birth order**		**Place of residence**		**Parental cohabitation/ marital status**		**Other individual based indicators**	
		HR^*^(95% CI)		HR^*^(95% CI)		HR^*^(95% CI)		HR^*^(95% CI)
**ALL DIAGNOSES COMBINED**								
(9)	**Number of full siblings**<**19 years**				**Cohabitation status**		
	0	1 (ref)			Alone	1 (ref)	
	1	1.12 (0.95–1.31)			Together	0.82 (0.69–0.99)	
	= >2	1.26 (1.03–1.53)					
(5)							**Rooms per person**	
							<1	1 (ref)
							1–1.25	0.76 (0.59–0.98)
							>1.25	0.80 (0.62–1.04)
							**Living space, tertiles**	
							Lower	1 (ref)
							Medium	0.78 (0.60–1.02)
							Upper	0.78 (0.60–1.03)
**HEMATOLOGICAL CANCERS**								
(9)	**Number of full siblings**<**19 years**				**Cohabitation status**		
	0	1 (ref)			Alone	1 (ref)	
	1	1.08 (0.81–1.44)			Together	0.92 (0.66–1.29)	
	= >2	1.18 (0.83–1.69)					
**LEUKEMIA**								
(5)							**Rooms per person**	
							<1	1 (ref)
							1–1.25	0.89 (0.55–1.43)
							>1.25	1.19 (0.76–1.87)
							**Living space, tertiles**	
							Lower	1 (ref)
							Medium	0.97 (0.59–1.58)
							Upper	1.01 (0.62–1.63)
**ALL**								
(12)			**Residential area**			
			Urban	1 (ref)		
			Mixed	1.16 (0.71-1.91)		
			Rural	0.88 (0.50–1.55)		
(13)	**Number of children**		**Place of living**		**Marital status**		
	Per increase of one child	0.99 (0.80–1.25)	Rural	1.08 (0.69–1.70)	Married	0.47 (0.27–0.83)	
			Semiurban	1.16 (0.74–1.81)	Other	1 (ref)	
			Urban	1 (ref)			
			**Travel distance (km) to hospital**				
			<50	1 (ref)			
			50–249	1.29 (0.80–2.10)			
			250+	1.24 (0.82–1.87)			
(14)	**Birth order**		**Place of residence**				
	1^st^	1 (ref)	Urban	1 (ref)			
	2^nd^	0.64 (0.37–1.10)	Mixed	1.12 (0.69–1.84)			
	3^rd^ and later	1.04 (0.55–1.95)	Rural	0.85 (0.49–1.49)			
	**Number of siblings**						
	0	1 (ref)					
	1	0.86 (0.48–1.52)					
	2	0.83 (0.42–1.67)					
	= >3	1.58 (0.73–3.44)					
(15)			**Distance from tertiary center**			
			Univariate				
			Short	1 (ref)			
			Long	1.05 (0.79–1.38)			
			Rurality				
			**Rurality**			
			Univariate				
			Urban	1 (ref)			
			Rural	1.15 (0.80–1.64)			
(20)	**Birth order**		**Place of residence**				
	1^st^	1 (ref)	Greater Copenhagen area	1 (ref)			
	2^nd^	1.05 (0.78–1.42)	Provincial cities	1.18 (0.88–1.59)			
	3^rd^	1.27 (0.85–1.89)	Rural areas	1.24 (0.81–1.91)			
	4^th^ and later	1.62 (0.85–3.09)	Peripheral rural areas	1.15 (0.55–2.40)			
	**Full siblings**						
	0	1 (ref)					
	1	1.05 (0.76–1.46)					
	2	1.19 (0.80–1.77)					
	= >3	1.31 (0.83–2.08)					
	**Full and half siblings**						
	0	1 (ref)					
	1	1.05 (0.71–1.55)					
	2	1.28 (0.82–1.98)					
	= >3	1.25 (0.76–2.05)					
**AML**								
(13)	**Number of children**		**Place of living**		**Marital status**		
	Per increase of one child	1.07 (0.69–1.66)	Rural	1.08 (0.48–2.46)	Married	0.83 (0.23–2.94)	
			Semiurban	0.52 (0.22–1.24)	Other	1 (ref)	
			Urban	1 (ref)			
			**Travel distance (km) to hospital**				
			<50	1 (ref)			
			50–249	0.84 (0.34–2.07)			
			250+	1.06 (0.48–2.31)			
(20)	**Birth order**		**Place of residence**				
	1^st^	1 (ref)	Greater Copenhagen area	1 (ref)			
	2^nd^	1.62 (1.01–2.59)	Provincial cities:	0.87 (0.54–1.40)			
	3^rd^	2.22 (1.13–4.34)	Rural areas	0.83 (0.45–1.55)			
	4^th^ and later	5.76 (2.01–16.51)	Peripheral rural areas	0.54 (0.18–1.63)			
	**Full siblings**						
	0	1 (ref)					
	1	1.11 (0.65–1.90)					
	2	1.09 (0.59–2.00)					
	= >3	2.27 (0.92–5.58)					
	**Full and half siblings**						
	0	1 (ref)					
	1	1.48 (0.79–2.75)					
	2	1.34 (0.67–2.67)					
	= >3	2.69 (1.11–6.52)					
**LYMPHOMA**								
(5)							**Rooms per person**	
							<1	1 (ref)
							1–1.25	0.88 (0.35–2.23)
							>1.25	0.35 (0.12–1.06)
							**Living space, tertiles**	
							Lower	1 (ref)
							Medium	0.61 (0.22–1.70)
							Upper	0.31 (0.08–1.11)
							
(20)	**Birth order**		**Place of residence**				
	1^st^	1 (ref)	Greater Copenhagen area	1 (ref)			
	2^nd^	0.97 (0.49–1.94)	Provincial cities	0.82 (0.41–1.63)			
	3^rd^	1.18 (0.41–3.40)	Rural areas	1.03 (0.38–2.78)			
	4^th^ and later	1.00 (0.20–5.11)	Peripheral rural areas	1.09 (0.23–5.17)			
	**Full siblings**						
	0	1 (ref)					
	1	1.06 (0.44–2.59)					
	2	2.26 (0.88–5.79)					
	= >3	0.91 (0.26–3.20)					
	**Full and half siblings**						
	0	1 (ref)					
	1	2.51 (0.63–9.92)					
	2	5.25 (1.40–19.70)					
	= >3	3.87 (0.92–16.31)					
**CNS TUMORS/TUMORS OF THE NERVOUS SYSTEM**								
(9)	**Number of full siblings**<**19 years**				**Cohabitation status**		
	0	1 (ref)			Alone	1	
	1	0.89 (0.67–1.18)			Together	0.70 (0.51–0.97)	
	= >2	1.03 (0.72–1.48)					
							
(5)							**Rooms per person**	
							<1	1 (ref)
							1–1.25	0.61 (0.39–0.97)
							>1.25	0.56 (0.34–0.92)
							**Living space, tertiles**	
							Lower	1 (ref)
							Medium	0.71 (0.43–1.17)
							Upper	0.61 (0.37–1.01)
							
(22)	**Birth order**		**Place of residence at diagnosis**		**Cohabitation status**		
	1st	1.0 (ref)	Greater Copenhagen area	1.0 (ref)	Living together	1 (ref)	
	2nd	0.97 (0.78–1.21)	Provincial cities	1.23 (0.98–1.56)	Living not together	1.07 (0.85–1.36)	
	3rd and later	1.00 (0.75–1.32)	Rural areas	1.38 (1.00–1.90)			
	**Full siblings**		Peripheral rural areas	1.17 (0.63–2.18)			
	0	1.0 (Ref)					
	1	1.12 (0.88–1.42)					
	2	0.98 (0.73–1.31)					
	= >3	0.87 (0.57–1.32)					
	**Children living in the household**						
	1	1.0 (Ref)					
	2	1.18 (0.91–1.52)					
	= >3	1.07 (0.79–1.44)					
							
(23)			**Driving distance to cancer center (miles)**				
			0–25	1 (ref)			
			26–50	0.97 (0.78–1.20)			
			>50	0.91 (0.76–1.11)			
**OTHER TUMORS**								
(9)	**Number of full siblings**<**19 years**				**Cohabitation status**		
	0	1 (ref)			Alone	1 (ref)	
	1	1.45 (1.11–1.89)			Together	0.80 (0.59–1.08)	
	= >2	1.29 (0.93–1.79)					
(24)			**Driving distance to cancer center (miles)**				
			<25	1 (ref)			
			25–50	1.1 (1.0–1.3)			
			>50	1.1 (1.0–1.3)			
							
(25)			**Driving distance to cancer center (miles)**				
			Univariate				
			<25	1 (ref)			
			25–49	0.6 (0.2–1.9)			
			> = 50	0.7 (0.2–2.0)			

* *Adjusted results if not otherwise stated. RR instead of HR is presented in some studies*.

*ALL, Acute lymphoblastic leukemia; AML, Acute myeloid leukemia; CI, Confidence interval; CNS, Central nervous system; HR, Hazard ratio*.

### All diagnoses combined

Combining all types of childhood cancer make the study population diverse but provides an overall pattern of potential inequalities. Four recent European register studies have looked at such associations. In Switzerland and Sweden, lower parental education was associated with higher mortality among children with cancer (5, 6), and a similar tendency was seen in Denmark (9). In Finland such an association was seen for the most recent years (7). An association between lower income and higher mortality was observed in Finland (7) and suggested in Denmark (9), but not found in Sweden (6). Furthermore, worse survival was observed for children with siblings, single parents, or poor living conditions (5, 9).

### Hematological malignancies

Hematological malignancies are the most common types of childhood cancer, and were also most frequently investigated regarding the association between SES and survival; 16 of the studies examined these diagnoses. In addition, one meta-analysis has been published (30), but due to its broader scope, the individual studies of relevance for this review will be discussed separately.

Various findings are reported regarding the association between parental SES and survival from hematological malignancies; while some studies found no association, others reported a gradient with lower survival among disadvantaged children, although the SES indicators of importance differed between studies. Overall, SES differences seemed to be less pronounced in hematological malignancies compared to childhood cancer overall. For leukemia and acute lymphoblastic leukemia (ALL), the associations with both parental education and income were inconclusive (5, 6, 12, 13). Disadvantaged parental SES, based on occupation, was associated with worse leukemia and ALL survival (10, 13), while no pattern was detected when the association between parental employment and survival was assessed in Finland (7). However, two studies reported point estimates suggesting an opposite gradient between parental education and survival from leukemia (5) and ALL (13), but these results were imprecise and not consistent between maternal and paternal education (5). Based on area-level indicators of SES, worse ALL and AML survival among children from low SES areas was observed in the US (16, 17, 19), also when insurance status was controlled for (16), while no association with event-free survival in ALL was seen in Canada (15). For lymphoma, higher parental education was suggested to be associated with better survival (5, 6), while findings for area-based SES indicators are inconclusive (5, 21).

An association between a larger number of siblings or higher birth order, and poorer survival from subtypes of hematological malignancies was suggested by studies conducted in Denmark (9, 20), while those pattern were not seen in Germany or Greece (13, 14).

Two US studies have looked at mortality close to a diagnosis of a hematological malignancy (11) or ALL (18). While one study reported an increased risk of death within the first month for children from lower SES neighborhoods (11), the other found no association between area-based income and inpatient mortality during the first period of chemotherapy (18).

### Tumors of the nervous system

The association between parental SES and survival after tumors of the nervous system were examined in seven of the included studies. Three studies suggest lower mortality among children of higher educated parents (5–7), while others did not find similar associations (9, 22). Individually measured parental income was assessed in three of the studies and these did not detect any statistically significant associations (6, 7, 9). Studies on other individually measured SES indicators suggested lower mortality among children of cohabitating parents (9, 22), or better living conditions (5), while no association with the number of siblings or birth order was found (9, 22). In addition, results of area-based indicators pointed toward an association between lower SES and higher mortality; in Texas children with the lowest SES-index had a higher risk of advanced stage disease and worse overall survival, although these associations were diluted in adjusted analyses (23). Another study from the US reported an association between several markers of disadvantaged SES areas and a higher risk of early deaths in CNS tumors, in univariate analyses (11). However, only poverty was included in the final adjusted model and the risk estimate was not reported (11).

### Other tumors

This section summarizes the findings for very diverse tumor types. Three studies investigated non-CNS solid tumors combined; a pattern of higher mortality among children of mothers with lower education was suggested (9), however, other indicators such as income and area-based SES-index did not show associations with mortality (9, 11, 24). Five of the studies were of small size or focused on cancer types with a very good survival which is reflected in the imprecise estimates and lack of statistical power (26–29). However, the point estimates in the majority of these studies were in the direction of lower survival among children of lower SES.

## Discussion

Findings of the 24 reviewed studies are diverse; some studies found no associations between socioeconomic or sociodemographic factors and survival while several indicated a social gradient with higher mortality among children from families of lower SES. When comparing the association within different cancer types, there is no clear suggestion of a particularly vulnerable subgroup, but hematological malignancies were most frequently investigated. Different indicators of SES appeared to be of importance in the studies which may indicate underlying mechanisms that vary between cancer types and health-care contexts, but can also be a result of diverse methodology, bias or random variation.

It has been acknowledged previously that different measurements of SES should not be understood as proxies for each other but instead they might have associations with health outcomes through different mechanisms (31). While income would indicate that economic resources of the family are of importance, education may reflect health literacy. However, our diverse findings do not clearly suggest a specific SES indicator of particular importance for childhood cancer survival. Parental education was more frequently investigated than income and also showed somewhat stronger associations; most often children of parents with lower education experienced higher mortality, however, there were also some findings pointing in the opposite direction but these were not statistically significant and not consistent. Only one study reported a statistically significant association between lower income and poorer survival (7), but point estimates in the other studies either pointed in the same direction, or were around the null value. These findings are very similar to the previous review by Gupta et al. (8).

### Potential mechanisms

The finding of poorer survival among children with lower parental SES requires further attention. Understanding the underlying mechanisms is the basis for any strategy to reduce health inequalities, but is a challenge since they likely differ between health-care setting and also childhood cancer types. Most studies focused on leukemia, and especially ALL, which does not necessarily reflect a particularly strong hypothesis connecting parental SES to survival from this cancer type, but might be the result of difficulties with statistical power in studies including more rare diagnoses. In fact, one of the studies found the strongest association for CNS tumors (5). A reason for this might be that, compared to leukemia, a low proportion of children with CNS tumors are treated within international standardized protocols in Switzerland (5). With less standardized protocols, there might be more room for influence from parents from higher SES, for example for referrals or second opinions, although this hypothesis has not yet been examined (5).

Another suggested mechanism is related to differences in how parents manage treatment adherence. The treatment of childhood cancer differs substantially between diagnoses, and the treatment strongly influences if the child will stay in hospital or at home. For example, treatment of ALL is long and a substantial part takes place at home where parents are usually responsible for the oral administration of drugs, see Lightfoot et al. (4) for a visualization. The results from the study by Lightfoot et al. demonstrated that SES differences in survival emerged during this period (4), which suggests that treatment adherence may be involved. This hypothesis is supported by other studies suggesting that higher SES, measured by different indicators, are associated with better treatment compliance (32–34), and compliance is of importance for treatment results in children with ALL (34, 35). In addition, when only inpatient mortality during induction chemotherapy was compared between children with ALL of different area-based income levels, no differences were observed (18). If parental responsibility for adherence to treatment was the main explanation of SES differences in survival, one would not expect any differences in mortality during inpatient treatment. With this reasoning one would also expect survival differences in ALL to be more pronounced compared to survival differences in AML, since AML is mainly treated within hospitals; however, included studies provide insufficient data to evaluate this hypothesis.

Not only have socioeconomic differences in childhood cancer survival been observed after a period of time, but also within the first month (11), and during the first year (6) after diagnosis. Possibly, early SES differences reflect differences in disease severity at diagnosis. Some of the studies have adjusted for this, but an association between SES and survival was still found (5, 10). When a potential association between SES and stage, or disease severity, at diagnosis has been assessed, some studies found no or very weak associations (10, 21, 23, 24, 26), while others indicated that children of lower SES may be more likely to have advanced disease (25, 27, 29).

Another potential explanation for socioeconomic survival differences might be related to differences in incidence of subtypes of cancers with different prognosis. Few of the studies have taken detailed subtype into account. However, Erdmann et al. (12) conducted a sensitivity analysis including only B-lineage ALL which resulted in similar conclusions as for all immunophenotypes of ALL combined, and Adam et al. (5) adjusted for histopathological group in their analysis of CNS tumors, which did not change their results.

### Methodology of reviewed studies

Several of the reviewed studies used register-based information which limits the risk of bias from non-participation and loss to follow-up. Most of the studies have identified their study population from cancer registers which also have been used by the International Agency for Research on Cancer for estimating cancer incidence (36, 37). Even if high registry coverage is even more important in incidence estimations, it is also important when assessing the association between social factors and survival. If the likelihood of being included in a study is associated with both SES and survival, biased results are obtained. However, such bias is not likely to have affected the conclusion of this review.

The source of information regarding social factors differed between studies, for example registers, birth certificates or questionnaires. One important aspect is, however, the temporality. Since a child's cancer diagnosis can affect some of the social factors, for example income, it is important that this information is collected before the diagnosis. All but one of the studies including individual measures of income assessed this before the child's cancer diagnosis. Income information in the study by Erdmann et al. (12) is based on interviews conducted within 2 years after a diagnosis, however, no association between family income and survival was found in this study. When area-based information is used, temporality is not that crucial since the child's diagnosis does not affect the income level in the neighborhood.

A general limitation with register-based studies is that they often are limited in terms of information on relevant confounders and mediators, such as severity of disease, treatment and adherence. As a result, several of the above discussed mechanisms are suggested but few are examined. Moreover, the choice of included SES indicators was seldom motivated in the reviewed studies.

Statistical power is weak in several of the studies, which reflects that the effect sizes are not very large, the overall prognosis is good and childhood cancer is rare. Different cancer types need to be considered separately due to diverse treatments and prognosis, however, this also decrease statistical power and studies on rare cancer types may not be able to detect potential socioeconomic differences. Of these reasons it is important to look at the direction and consistency of findings rather than only statistical significance. This is also important when interpreting the results of studies using area-based indicators of SES. As previously acknowledged, e.g., (10, 15), using area-based measures of SES as proxies for individual measurements can lead to ecological fallacy, a non-differential exposure misclassification which might dilute an association should one exist.

Time period of diagnosis differed greatly between studies. Studies focusing on recent periods have lower statistical power due to limited number of included children and increased survival rates. However, the association between parental SES and survival may have changed with calendar time; e.g., Njoku et al. (10) included children diagnosed 1968-2010 and showed a tendency of less SES differences during the latest years. However, focusing on more recent time periods, Tolkkinen et al. (7) found differences in survival according to parental education primarily in children diagnosed during 2000–2009, compared to in the 1990's.

Another time aspect is the differences in follow-up time between the included studies. While a few studies assessed mortality closely after the cancer diagnosis, most of the studies focused on mortality up to 5 or 10 years. Comparisons between these two types of studies should be done with caution since the mechanisms behind potential SES differences in mortality directly at time of diagnosis and several years after are probably very different.

### Strengths and limitations

This review was based on an extensive literature search and includes studies of several indicators of SES and their associations with survival from different types of childhood cancer. The search strategy and study selection are described in detail to ensure reproducibility. Moreover, descriptions of included studies and relevant results are shown in detail to visualize the diversity. Since the choice of SES indicators, definition of study population, and adjustment variables differed to such extent between studies a comparison of effect estimates is hampered (8).

Some limitations with this review need to be acknowledged. Only one data source (PubMed) was used to identify studies; potential articles searchable only in databases other than PubMed are therefore not included. However, in the field of childhood cancer epidemiology we find it unlikely that significant articles are not identified in PubMed. Another limitation is that no formal bias assessment was performed. However, the methodology of included studies are described in Table 1 for transparency, and commented in the above section. In addition, we cannot rule out that some publication bias may be present, i.e., that studies showing no associations are less likely to be published. In such case, the conclusions from our review may be too strong regarding the association of low SES and worse childhood cancer survival.

## Conclusion

This review has summarized the most recent publications on the association between parental SES and childhood cancer survival in high-income countries. Even though some of the reviewed studies found no differences in survival between children from diverse socioeconomic backgrounds, worse survival among children of lower SES were observed for several cancer types, contexts, and SES indicators. Studies that more carefully investigate specific underlying mechanisms for the socioeconomic differences in survival are lacking. Collaborative studies are needed to increase statistical power to enable investigation of the association within homogenous cancer types which will increase the understanding of the mechanisms involved, and allow targeted interventions to reduce health inequalities.

## Author contributions

HM, KM, FE, GT, MH, and MF contributed to the design of the study. HM screened titles, abstracts and full-texts. HM drafted the manuscript. HM, KM, FE, GT, MH, and MF reviewed the manuscript and approved the final version.

### Conflict of interest statement

The authors declare that the research was conducted in the absence of any commercial or financial relationships that could be construed as a potential conflict of interest.
