# Supplementary material for: Survival After Childhood Cancer–Social Inequalities in High-Income Countries
Source: Front Oncol. 2018 Oct 31;8:485. doi: 10.3389/fonc.2018.00485 (PMC6238081; doi:10.3389/fonc.2018.00485)
Supplement: Supplementary file 1 [file Data_Sheet_1.PDF]

Supplementary material

Survival after Childhood Cancer

–Social Inequalities in High-income Countries

Hanna Mogensen*, Karin Modig, Giorgio Tettamanti, Friederike Erdmann, Mats Heyman, Maria Feychting

*** Correspondence:** Hanna Mogensen: hanna.mogensen@ki.se

Supplementary Table 1. PubMed search query

**Cluster**	**Search terms**
**A: Cancer**	“neoplasms/epidemiology”[MeSH] OR cancer[Title/Abstract] OR oncology[Title/Abstract] OR malignancies[Title/Abstract] OR malignancy[Title/Abstract] OR tumor[Title/Abstract] OR tumors[Title/Abstract] OR tumour[Title/Abstract] OR tumours[Title/Abstract] OR leukemia[Title/Abstract] OR leukaemia[Title/Abstract] OR “CNS tumors”[Title/Abstract] OR “CNS tumours”[Title/Abstract] OR lymphoma [Title/Abstract]
**B: Survival**	Survival[MeSH] OR mortality[MeSH] OR prognosis[MeSH] OR "neoplasms/mortality"[MeSH] OR Survival[Title/Abstract] OR mortality[Title/Abstract] OR prognosis[Title/Abstract]
**C: Children and adolescents**	Child[Mesh] OR Adolescent[Mesh] OR Pediatrics[Mesh]) OR children[Title/Abstract] OR childhood[Title/Abstract] OR adolescents[Title/Abstract] OR adolescence[Title/Abstract] OR paediatric[Title/Abstract] OR pediatric[Title/Abstract] OR teen[Title/Abstract]
**D: Socioeconomic and sociodemographic factors**	1: "Socioeconomic Factors"[Mesh] OR "Social Conditions"[Mesh] OR "Educational Status"[Mesh] OR "Employment"[Mesh] OR "Income"[Mesh] OR "Occupations"[Mesh] OR “marital status"[Mesh] OR “family characteristics” [Mesh] OR “social class”[Mesh] OR “socioeconomic status”[Title/Abstract] OR “socioeconomic factors”[Title/Abstract] OR “socioeconomic position”[Title/Abstract] OR “sociodemographic factors”[Title/Abstract] OR “family characteristics”[Title/Abstract] OR “family

	circumstances"[Title/Abstract]
	2: educational[Title/Abstract] OR "SES neighborhoods"[Title/Abstract] OR education[Title/Abstract] OR employment[Title/Abstract] OR occupation[Title/Abstract] OR unemployment[Title/Abstract] OR profession[Title/Abstract] OR work[Title/Abstract] OR job[Title/Abstract] OR income[Title/Abstract] OR "economic status"[Title/Abstract] OR cohabitation[Title/Abstract] OR "marital status"[Title/Abstract] OR married[Title/Abstract] OR "place of residence"[Title/Abstract] OR "living area"[Title/Abstract] OR sibling[Title/Abstract] OR siblings[Title/Abstract] OR birth order[Title/Abstract] OR residence[Title/Abstract] OR rural[Title/Abstract] OR distance[Title/Abstract]
	1 AND 2
**E: Restriction of publication year**	"2012/12/01"[PDat] : "2018/06/15"[PDat]
**Final**	A AND B AND C AND D AND E (333 articles in total)
